# RUNX1 and breast cancer

**DOI:** 10.18632/oncotarget.17249

**Published:** 2017-04-19

**Authors:** Jose Mercado-Matos, Asia N. Matthew-Onabanjo, Leslie M. Shaw

**Affiliations:** Department of Molecular, Cell and Cancer Biology, University of Massachusetts Medical School, Worcester, MA, USA

**Keywords:** breast cancer, E-Cadherin, estrogen receptor, EMT, RUNX1

RUNX1 is a member of the RUNX family of transcription factors that also includes RUNX2 and RUNX3 [1]. Early studies identified RUNX1 as a key regulator of definitive hematopoiesis and the role of RUNX1 as a tumor suppressor in leukemia is well established [1]. In more recent sequencing studies of solid tumors, molecular alterations have been identified in other cancer types, suggesting a broader role for RUNX1 in epithelial cells and carcinomas. RUNX1 is the predominant RUNX protein expressed in the normal human breast epithelium and it is the only RUNX family member for which somatic mutations have been identified in human breast cancer. These mutations are primarily loss of function mutations that occur through nonsense, frameshift or missense mutations within the Runt DNA-binding domain [1]. These RUNX1 mutations occur almost exclusively in the ER+, luminal subtype of breast cancer and indicate a tumor suppressor role for RUNX1 [2]. In contrast, elevated levels of RUNX1 expression correlate with poor outcomes in triple negative breast cancers (TNBCs), indicating a positive, oncogenic role for RUNX1 in this breast cancer subtype [3]. These disparate findings support a cell-context dependent role for RUNX1 in breast cancer.

The functional consequences of RUNX1 loss or gain of expression that contribute to breast cancer development and progression have not been fully elucidated. A role for RUNX1 in suppressing ER oncogenic signaling is suggested by the fact that combined loss of Runx1 with either TP53 or Rb1 leads to hyperproliferation of ER+ luminal cells in the mouse mammary gland [2]. Inactivating mutations of RUNX1 may be an early event that enhances estrogen signaling, creating a permissive environment for the development of ER+ luminal tumors. Regulation of ER signaling would explain the luminal subtype specificity of RUNX1 mutations in human breast cancer. With regard to tumor progression, the first evidence to support an association of RUNX1 with cancer metastasis came from a gene expression analysis comparing human primary adenocarcinomas of multiple types with unmatched metastatic lesions [4]. A 17-gene expression signature that distinguished primary from metastatic adenocarcinomas, and that identified primary tumors that were most likely to be associated with metastasis and poor clinical outcomes, included RUNX1 (downregulated expression). A recent study by Hong et al provides a mechanistic explanation for this association of RUNX1 loss with tumor progression to metastasis [5]. Specifically, TGF-β downregulates RUNX1 mRNA and protein expression in the normal, immortalized mammary epithelial cell line, MCF-10A, and loss of RUNX1 is required for the ability of TGF-β to induce an epithelial mesenchymal transition (EMT) in these cells. Importantly, loss of RUNX1 expression alone promotes EMT, which is also observed for ER+, luminal MCF-7 cells, indicating a direct role for RUNX1 in maintaining the epithelial phenotype. One mechanism by which RUNX1 inhibits EMT is through interactions with the *CDH1* promoter to positively regulate E-Cadherin expression.

In apparent conflict with the role of RUNX1 as a suppressor of EMT in ER+ luminal tumors, downregulation of RUNX1 expression in TN tumors inhibits tumor invasion and metastasis [6]. How do both high and low levels of RUNX1 expression contribute to tumor progression? Although loss of E-Cadherin is commonly associated with the mesenchymal, invasive phenotype that occurs with EMT, tumor cells can also rely upon E-Cadherin expression for collective invasion from the tumor [7]. Moreover, in a recent study investigating the role of cancer associated fibroblasts (CAFs) in tumor invasion, a heterotypic interaction between N-Cadherin on CAFs and E-Cadherin on tumor cells was required for efficient invasion of squamous cell carcinoma (SCC) cells [8]. Therefore, either high or low levels of RUNX1 could regulate invasion and promote tumor progression in breast cancer through a mechanism involving regulation of E-Cadherin expression.

In summary, increasing evidence supports a role for RUNX1 in breast cancer progression. Understanding how RUNX1 functions in a cell context-dependent manner will be essential for determining the mechanism(s) by which this transcription factor impacts tumor biology.
